# Uncovering Mechanisms of NRTI Induced Cellular Senescence

**DOI:** 10.1093/geroni/igaf122.3865

**Published:** 2025-12-31

**Authors:** Niharika Kura, Chisaka Kuehnemann, Gailan Constantino, Christopher Wiley

**Affiliations:** Tufts University, Boston, Massachusetts, United States; Tufts University, Boston, Massachusetts, United States; Tufts University, Boston, Massachusetts, United States; Jean Mayer HNRCA at Tufts University, Boston, Massachusetts, United States

## Abstract

People living with HIV (PLWHIV) currently have extended lifespans, but experience increased age-related diseases and younger ages compared to their HIV negative counterparts. Nucleotide reverse transcriptase inhibitors (NRTIs) are breakthrough medications at the center of HIV therapy. However, these drugs have been known to cause side effects associated with premature aging, Early generation NRTIs, such as thymidine analogs AZT and d4T, caused lipodystrophy and myopathies, while modern NRTIs, such as the adenosine analogs TDF and TAF, cause renal toxicity and dyslipidemia. All of these drugs induce senescence in vitro; however, their mechanism of senescence induction is distinct. In this study, we show that early generation NRTIs cause senescence via mitochondrial dysfunction, whereas modern NRTIs cause senescence via genotoxic stress. This study demonstrates potential cellular mechanisms associated with accelerated aging seen in PLWHIV, as well as potentially linking methods of senescence induction to clinical presentations of aging pathologies.

